# Balancing energy resilience and mobility: a multi-objective strategy for deploying shared autonomous electric vehicles during power outages

**DOI:** 10.1038/s44333-026-00081-9

**Published:** 2026-02-16

**Authors:** Jônatas Augusto Manzolli, Jiangbo Yu, Alessandro Vissarios D’Apice, Luis Miranda-Moreno

**Affiliations:** 1 Department of Civil Engineering, McGill University, Montreal, QC Canada; 2 INESC Coimbra, Department of Electrical and Computer Engineering, University of Coimbra, Coimbra, Portugal

**Keywords:** Energy grids and networks, Science, technology and society, Operational research

## Abstract

As cities face increasing climate-induced disruptions, shared autonomous electric vehicles (SAEVs) emerge as a dual-purpose solution to sustain passenger mobility and provide emergency power. This study introduces a framework that combines GIS-based spatial analysis and multi-objective optimization to evaluate SAEV fleet performance during power outages. Using Montreal as a testbed, we simulate the operations of a mid-sized SAEV fleet under varying power disruption scenarios. Our analysis reveals a critical operational trade-off: the fleet can meet up to 2% of daily mobility demand or supply 28% of energy needs in affected zones, but not simultaneously. The sensitivity analyses indicate that improving charging infrastructure yields greater operational benefits than increasing battery capacity. Further, revenue from energy provision increases significantly with larger fleets and higher charger power. The findings underscore the importance of coordinated infrastructure planning and incentive design to enable SAEVs to effectively support transport continuity and urban energy resilience.

## Introduction

**Fig. 1:**
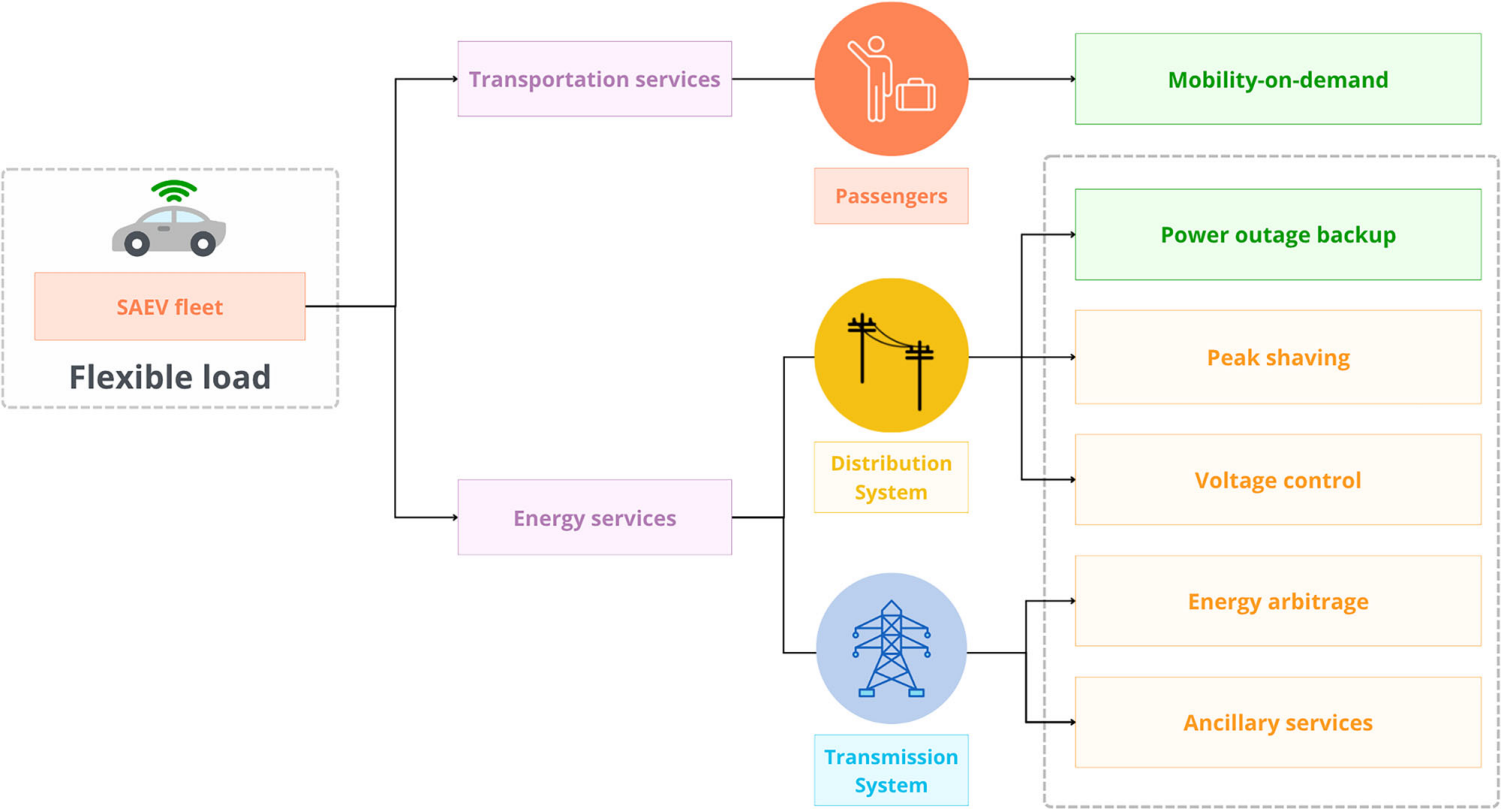
Mobility and energy services offered by SAEVs.

The escalating impacts of climate instability demand robust mobility solutions that enhance the resilience of broader urban systems^1,2^. Among the most promising advances are shared autonomous electric vehicles (SAEVs), which combine on-demand mobility with the ability to support the grid by utilizing batteries as mobile storage^3^. SAEVs overcome the limitations of human-driven, privately owned electric vehicles (EVs), such as limited hours, breaks, and compliance issues, making them valuable as mobile energy storage systems that can support future energy frameworks by offering services to distribution system operators (DSOs). Figure 1 presents different services with which SAEVs could be engaged (highlighted in green are the services assessed in the context of this paper).

More specifically, SAEVs can “transport” energy from standard operation zones to areas experiencing outages. Their autonomous design enables them to accurately follow dispatch commands, ensuring quick and reliable responses during emergencies. This approach enhances emergency logistics, reduces response times, and fosters a resilient and adaptable urban transportation system.

However, the primary goal of an SAEV operator (SAEV-O) is to provide transportation, reducing customer wait times and fleet relocation for charging. In such cases, fleets would likely not serve as backups during power outages, as this would hinder mobility-on-demand services. Conversely, offering grid services could boost SAEV-O revenues, creating additional income. To balance these conflicting objectives, we propose a multi-objective optimization (MOO) program that evaluates (i) maximizing revenues during disruptions through grid services and (ii) improving transportation by lowering passenger wait times. We suggest using an ε-constraint method and including a Montreal case study to obtain reliable solutions. While research on SAEVs and grid integration has gained traction recently^4,5^, few existing studies consider the synergistic effect of flexible ownership, automation, and electrification on urban and energy resilience during outages. To fill this gap, we evaluate the following questions:

1. (Operational feasibility) Is it feasible for an SAEV-O to contribute to the stability of the power grid while meeting regular travel demand?
2. (Service level impact) What are the operational impacts on the transportation service level when reallocating part of the fleet in case of an outage event?
3. (Financial attractiveness) What are the impacts on a SAEV-O’s revenues when offering energy to the grid?

This research aims to deepen the understanding of urban mobility, energy resilience, and technological innovation by exploring these questions. It provides insights for policymakers, urban planners, and scholars working toward sustainable cities. To the authors’ knowledge, no study has employed an MOO approach to address the conflicting objectives of SAEV-Os in power grid resilience. Therefore, the main contributions of this paper are:

- This paper proposes an MOO approach to address the conflicting objectives of SAEV-Os in transportation and grid services.
- The paper presents a comprehensive case study of Montreal, providing a diverse evaluation of the proposed application’s effectiveness and practical implications in a real-world urban setting.

The organization of this paper is as follows. The Literature Review section discusses the roles of autonomous vehicles (AVs) and EVs in urban infrastructure resilience as well as optimization modeling techniques. The Methods section presents a framework for SAEV integration, modeling, and optimization. The Case Study section describes the datasets used for the assessment in Montreal. The Results section discusses the findings and applications of the optimization experiments. The Conclusions section summarizes the paper, offering insights and directions for future research in urban mobility.

### Literature review

This review combines two key elements that support our study. First, we gather evidence on how electric, autonomous, and shared vehicles boost urban resilience through flexible, centrally managed mobility and portable energy storage. Second, we review optimization and control models for SAEV fleets, focusing on approaches that integrate mobility operations with grid services.

### Roles of shared, autonomous, and electric vehicles in urban resilience

Urban resilience refers to a city’s ability to absorb, adapt to, and recover from disruptive events while maintaining its well-being. It arises from the interaction of infrastructure, institutions, and social systems that confront hazards ranging from extreme weather to technological failures^6^. As Cutter et al. note^7^, resilience drivers vary by context: in urban areas, economic capital and networked services are dominant, while in rural settings, community capital, social cohesion, and trust play a larger role. This distinction underscores the need for strategies tailored to dense, service-rich environments.

In this context, shared mobility enhances resilience by providing an on-demand fleet that can be rapidly re-tasked without relying on private owners. Because vehicles are pooled and centrally coordinated, operators can reallocate supplies to critical corridors, sustain essential trips, and support evacuation or delivery operations during emergencies, thereby improving service continuity and reducing response times^8^. Autonomy adds reliability, coverage, and safety to this response. AVs can follow system-level directives, operate continuously without duty-cycle constraints, and maintain service when human driving would be risky or infeasible. Networked sensing and communication enable AVs to stabilize flows, reroute around blockages, and execute prioritized missions (e.g., medical access, supply runs) with reduced crash risk and faster clearance^9^. Electrification complements these capabilities by decoupling vehicles from liquid-fuel logistics and enabling mobile energy storage. EVs can provide backup power to critical loads during grid outages and participate in vehicle-to-grid (V2G) services that supply electricity during peak periods or emergencies, thereby enhancing energy security and flexibility^10,11^. Combining these three dimensions yields SAEVs, a platform with a distinctive resilience value^3,12^. SAEVs combine mobility and electrical capacity, offering resilience in urban areas by operating autonomously or following commands, and providing transport and energy during disruptions. Their design affects robustness and response, supporting missions beyond energy supply shortages, such as evacuation or medical logistics, underscoring the need for careful orchestration across multiple sources^13^.

### Modeling, evaluation, and optimization techniques

Research on SAEV operations and their coupling with power systems has progressed along two complementary tracks. One leverages agent-based modeling to capture heterogeneous traveler behavior and fleet interactions, following classic formulations^14^. The other advances optimization and control methods that embed system dynamics and, increasingly, real-time data, to support operational decision-making at scale (which are the focus of this review). Most contributions prioritize the operational efficiency of SAEVs^15^. Liao et al.^16^ examine the economic and environmental effects of integrating V2G into mixed-range SAEV fleets, showing that V2G can reduce operating costs and emissions while explicitly accounting for battery degradation and range anxiety. Dean et al.^17^ use an agent-based model to co-optimize charging and repositioning, reporting sizable improvements in service quality. Zhang et al.^18^ develop a model predictive control (MPC) scheme that solves a mixed-integer linear program (MILP) at each step, demonstrating real-time feasibility for moderate-sized systems. Iacobucci et al.^19^ apply MPC to jointly optimize routing/relocation and V2G charging, reducing charging costs without materially increasing waiting times, especially when electricity prices are volatile. Melendez et al.^20^ formulate a robust MILP for a cyber-physical system of SAEVs and charging hubs, linking day-ahead commitments with real-time price fluctuations and demand uncertainty to coordinate power and mobility decisions. Qi et al.^21^ propose an analytics framework for urban microgrids, showing that expanding SAEV fleets can raise solar-powered microgrid self-sufficiency and resilience in New York City. Closer to disruption response, Amirioun et al.^22^ design a scheduling scheme in which DSOs pre-negotiate contracts and issue requests to SAEVs to restore critical loads after extreme events, allowing fleets to serve passengers while meeting energy delivery deadlines. Liu et al.^12^ develop a dynamic optimization model for vehicle-to-building (V2B) support of critical facilities, finding that SAEVs can supply emergency power with acceptable impacts on passenger waiting times.

### Gap and contribution

Although research on SAEV operations and grid integration is substantial, most studies optimize either mobility performance or grid support, treating the two objectives in parallel rather than within a single decision space. As a result, three practical questions remain largely unanswered: (i) how much grid support can be provided without unacceptable losses in passenger service during outages; (ii) whether reassigning fleets is economically feasible once battery degradation costs are counted; and (iii) what remuneration rules make participation rational for operators. Our work addresses this gap by developing an MOO model that simultaneously assesses passenger demand fulfillment and transportation revenue, while also reducing outages, explicitly accounting for battery capacity fade, and connecting discharge to a tariff-based remuneration mechanism. This produces an actionable Pareto frontier that makes the mobility–energy trade-off transparent and directly usable for policy (e.g., setting tariff floors and service guardrails). Table 1 summarizes the contrast with prior studies, showing that, to our knowledge, the proposed framework is the first to integrate all five elements, namely passenger demand, transportation revenue, outage mitigation, battery degradation, and grid-service remuneration.

**Table 1:** Comparison of the literature with our study

Ref.	Approach	Passenger demand	Transportation revenue	Outage mitigation	Battery degradation	Grid services remuneration
Liu et al.^12^	Dynamic optimization	✓	✓	✓	-	-
Liao et al.^16^	Simulation	-	-	-	✓	✓
Dean et al.^17^	Agent-based approach	✓	-	-	-	-
Zhang et al.^18^	MPC	✓	-	-	-	-
Iacobucci et al.^19^	MPC	✓	-	-	✓	✓
Melendez et al.^20^	Stochastic optimization	✓	✓	-	-	✓
Qi et al.^21^	Simulation	-	-	✓	-	-
Amirioun et al.^22^	Simulation	-	-	✓	-	✓
Our Proposal	MOO	✓	✓	✓	✓	✓

We recognize that the energy transition is still underway, and our analysis is intentionally forward-looking, considering a plausible future in which SAEVs and V2G-capable neighborhood charging are widely available. At the same time, many large pilots are already pointing in this direction. Ongoing AV and V2G demonstrations across China, Europe, and the USA show increasing operational maturity^23–27^. For example, Baidu’s Apollo Go reports service expansion, with over 1000 robotaxis operating in ~15 cities across China by mid-2025^28^. These developments narrow the gap between our future scenario and current practice. Our goal is to explore how transportation and energy systems can be integrated under such conditions so that today’s policies, investment decisions, tariff structures, funding for bidirectional neighborhood hubs, and operational safeguards are prepared as scale is achieved.

## Method

**Fig. 2:**
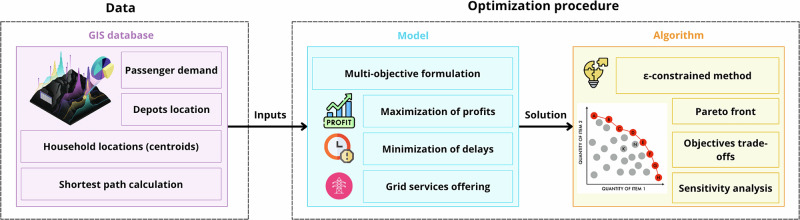
Methodological framework.

Figure 2 summarizes our three-step framework. First, we build a GIS database with origin-destination (OD) demand, household and destination locations, and depot sites. Second, we formulate an MOO model that balances cost, service, and grid support using that data. Third, we solve the model with an ε-constraint sweep to trace trade-offs and identify efficient fleet capacities and operations. The following subsections describe each step.

### GIS database component

The GIS database underpins the framework by enabling spatial analysis, routing, and basic validity checks. It consolidates the spatial and attribute layers needed to evaluate and optimize SAEVs for urban mobility and energy resilience. Key data types stored in the database include:

- **OD demand data:** Spatial and hourly demand by zone, including origins, destinations, passenger counts, and departure times aggregated to tracts or grid cells.
- **Origin and destination locations:** Centroid representations of households and activity sites are used to assess SAEV demand and the spatial distribution of energy needs during disruptions.
- **Road network data:** Node–edge topology with attributes for routing and travel-time estimation to optimize SAEV routes and service areas.
- **Charging and discharging infrastructure locations:** Existing and candidate sites used to plan operations and balance mobility service with energy provision during outages.
- **Outage zones:** Spatial layers identifying outage-prone or active outage areas to model where SAEVs can supply backup energy.

The GIS database is structured to efficiently store and manage large volumes of spatial and attribute data, facilitating the integration of multiple data sources. The database is typically organized into layers, each representing a specific data type (e.g., OD demand, road network, charging locations). The data were stored in formats compatible with GIS software, such as shapefiles, geodatabases, or spatial databases like PostgreSQL with PostGIS extensions.

### MOO programming problem formulation

The optimization model builds upon previous works^12,18–20^. However, our approach differs from the literature by expanding the previous studies in three main aspects. First, unlike later studies, we propose an MOO approach to evaluate the trade-off between maximizing revenue and enhancing ride services from the SAEV-O’s perspective. Therefore, the two objective functions aim to maximize profit and enhance transportation services. Second, unlike other models in which vehicles were immediately assigned when an outage occurred, in our formulation, vehicles provide energy services to the grid only if it is economically profitable. We believe this approach may be more realistic for prospective scenarios. Lastly, we incorporate a more detailed evaluation of battery degradation costs, as introduced in our previous work^29^. As aforementioned, it is unlikely that the fleets would be quickly assigned to provide these grid services, since the primary objective of an SAEV-O is to deliver riding services, unless an economic compensation better than the estimated riding prices is offered. Therefore, this optimization problem has conflicting objectives regarding transportation service levels and the provision of grid services. In this context, we made the following assumptions to streamline the problem further:

- The SAEV-O follows a station-based business model, in which vehicles are located at fixed rental locations, ensuring they can always return to their parking spot after a trip.
- To meet charging infrastructure needs, we use a business model similar to that of leading shared fleet operators (e.g., Communauto in Montreal). In this context, a robust combination of proprietary and public charging points underpins the EV fleet’s operations. Accordingly, we assume that recharging is possible whenever a vehicle is parked.
- The affected zones are prepared to receive energy from the SAEVs, i.e., they have bidirectional charging spots.
- During the operation, an average speed is considered to estimate the average travel time and energy consumption from two nodes.

Also, the optimization model’s nomenclature is presented in Table 2.

**Table 2:** MOO model nomenclature

Sets and indexes	
$$i,j$$	Index of nodes for pick-ups and drop-outs.
$$s$$	Index of nodes of parking (and charging) spots.
$$o$$	Index of nodes of power outage locations.
$$k$$	Index of vehicles.
$$t$$	Index of timesteps.
$${\mathscr{N}}$$	Set of all nodes in the network, $${\mathscr{N}}=\{1,\ldots ,N\}$$.
$${\mathcal{A}}$$	Set of all arcs in the network, $${\mathcal{A}}{\mathscr{\subseteq }}{\mathscr{N}}\times {\mathscr{N}}$$.
$${\mathcal{S}}$$	Subset of nodes where parking/charging spots are located, $${\mathcal{S}}=\{1,\ldots ,S\}$$.
$${\mathcal{O}}$$	Subset of nodes where outages occur, $${\mathcal{O}}=\{1,\ldots ,O\}$$.
$${\mathcal{K}}$$	Set of vehicles, $${\mathcal{K}}=\{1,\ldots ,K\}$$.
$${\mathcal{T}}$$	Set of timesteps, $${\mathcal{T}}=\{1,\ldots ,T\}$$.
**Parameters**	
$${B}_{k}$$	Battery capacity of vehicle k, [kWh].
$${P}_{i,j,t}$$	Number of passengers arriving at node i with destination j at time t, [People].
$${\pi }^{{ch}}$$	Price to buy energy (grid), [CA$/kWh].
$${\pi }^{{out}}$$	Price to sell energy (outage), [CA$/kWh].
$${\pi }^{{trip}}$$	Price paid by each customer per trip, [CA$/passenger]
$${E}_{o,t}^{{req}}$$	Energy required in an affected zone o per timestep t, [kWh].
$${\gamma }^{\max }$$	Maximum state-of-charge, [%].
$${\gamma }^{\min }$$	Minimum state-of-charge, [%].
$${\gamma }_{k}^{0}$$	Initial state-of-charge for each vehicle k, [%].
$${\tau }_{i,j}$$	Travel time between node i to node j, [Minutes].
$$\xi$$	Energy consumption rate, [kWh/km].
$${\rho }^{{ch}}$$	Charging rate (parking spots), [kW].
$${\rho }^{{out}}$$	Discharging rate (outage location), [kW].
$$\eta$$	Charger efficiency, [%].
$${R}^{{bat}}$$	Battery replacement costs, [CA$/kWh].
$${N}^{{Cy}}$$	Number of cycles until end-of-life, [Cycles].
$${\lambda }^{{DoD}}$$	Depth of discharge, [%].
**Variables**	
$${x}_{k,i,j,t}$$	Binary variable indicating if vehicle k is carrying passengers from node i to node j at timestep t {0,1}.
$${y}_{k,i,j,t}$$	Binary variable indicating if vehicle k is reallocating (empty) from node i to node j at timestep t {0,1}.
$${z}_{k,i,t}$$	Binary variable indicating if vehicle k is parked at node i at timestep t {0,1}.
$${p}_{k,i,t}$$	Binary variable indicating if vehicle k is arriving at node i at timestep t {0,1}.
$${u}_{k,s,t}^{{ch}}$$	Binary variable indicating if vehicle k is charging at node s at timestep t {0,1}.
$${u}_{k,o,t}^{{out}}$$	Binary variable indicating if vehicle k is offering energy in affected zone o at time t {0,1}.
$${d}_{i,j,t}$$	Number of passengers waiting at node i with destination j at timestep t.
$${e}_{k,t}$$	Energy of vehicle k at timestep t [kWh].
$${g}_{k,s,t}^{{ch}}$$	Energy charged from vehicle k at node s at timestep t [kWh].
$${g}_{k,o,t}^{{out}}$$	Total energy offered from vehicle k to affected zone o at timestep t [kWh].
$${a}_{k}$$	Total energy taken from a vehicle k battery through its lifespan [kWh]
$${\omega }_{k,t}$$	Battery degradation costs of vehicle k in timestep t to offer energy to the grid [CA$].

### Objective functions

**Fig. 3:**
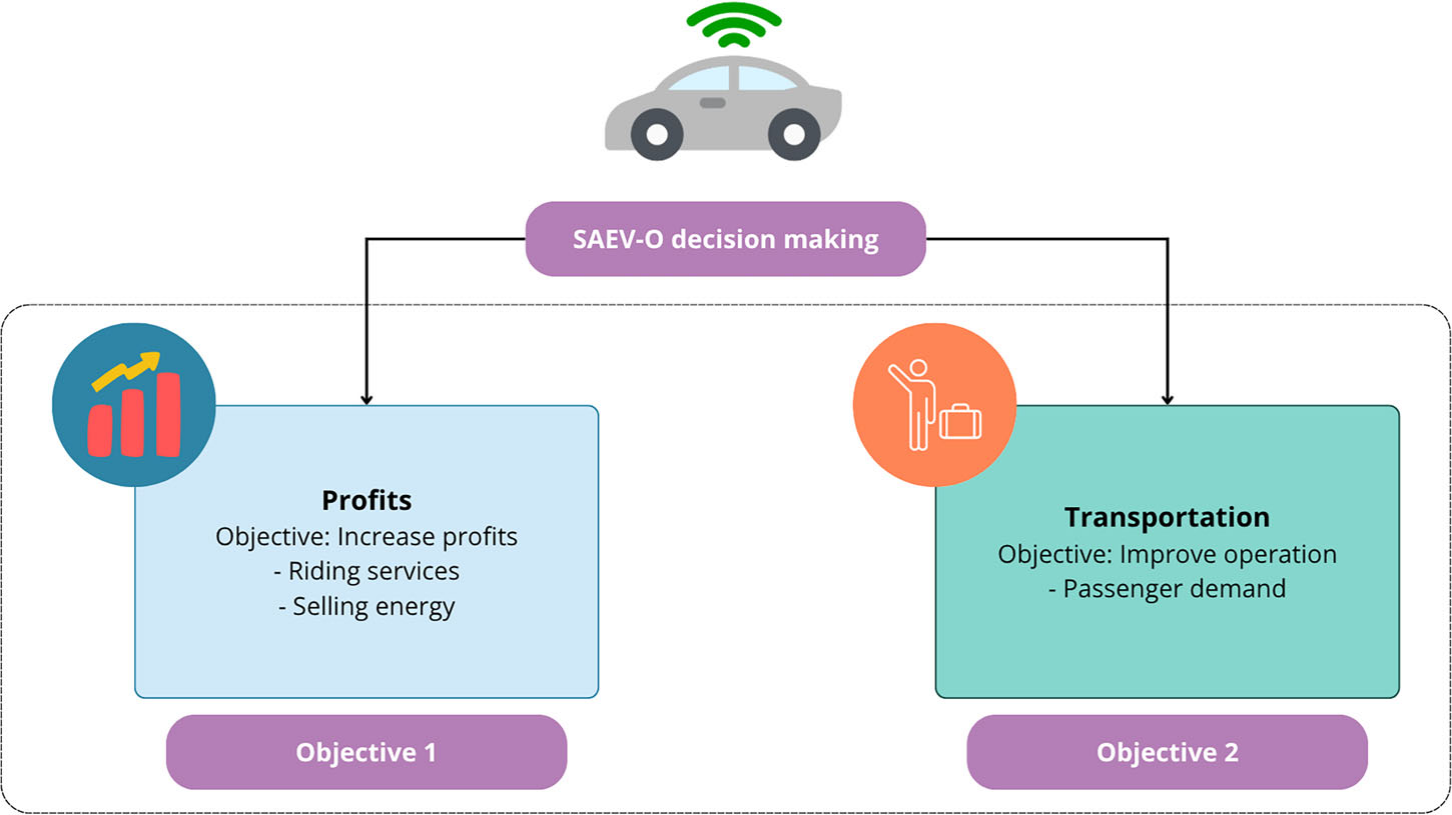
SAEV-O decision-making process.

This framework considers two distinct goals (Fig. 3): (a) the first focuses on the strategic allocation of the fleet to perform riding services or offer energy to the grid, aiming to maximize the profits of the SAEV-O; (b) the second deals with the operational tactics of the SAEVs, ensuring optimal responses to fluctuating passenger demands, aiming to improve the overall transportation level of the fleet.

To accommodate the trade-offs concerning maximizing profits and improving the transportation service, we developed two distinct objective functions (profits and riding service quality), which are detailed in the following.

- **Profits:** We split the profit maximization into two different terms, which are described in Eqs. (1) and (2).

1 $${f}_{t}^{{trip}}=\mathop{\sum }\limits_{k{\mathscr{\in }}{\mathcal{K}}}\mathop{\sum }\limits_{\left(i,j\right){\mathscr{\in }}{\mathcal{A}}}{\pi }^{{trip}}.{x}_{k,i,j,t},\forall t{\mathscr{\in }}{\mathcal{T}}$$

2 $${f}_{t}^{{eng}}=\mathop{\sum }\limits_{k{\mathscr{\in }}{\mathcal{K}}}\left(\mathop{\sum }\limits_{s{\mathscr{\in }}{\mathcal{S}}}{g}_{k,s,t}^{{ch}}.{\pi }^{{ch}}-\mathop{\sum }\limits_{o{\mathscr{\in }}{\mathscr{O}}}{g}_{k,o,t}^{{out}}.{\pi }^{{out}}+{\omega }_{k,t}\right)\,,\forall t{\mathscr{\in }}{\mathcal{T}}$$

Equation (1) determines the revenues stemming from offering riding services. Equation (2) evaluates the costs (or profits) generated by the energy transactions of the fleet. We consider the energy charged (${g}_{k,t}^{{ch}}$), the energy offered during outages (${g}_{k,t}^{{out}}$), and the battery degradation costs (${\omega }_{k,t}$) due to returning energy to the grid. Equation (3) presents the first objective function, which aims to maximize the revenues of the SAEV-O.

3 $$\max {f}_{1}=\mathop{\sum }\limits_{t{\mathscr{\in }}{\mathcal{T}}}\left({f}_{t}^{{trip}}-{f}_{t}^{{eng}}\right)$$

**b) Riding services quality:** Eq. (4) presents the second objective function, which aims to minimize number of passengers (${d}_{i,j,t}$) that are waiting for a ride, and by this mean, improving the transportation service level to the customers.

4 $$\min {f}_{2}=\mathop{\sum }\limits_{t{\mathscr{\in }}{\mathcal{T}}}\mathop{\sum }\limits_{\left(i,j\right){\mathscr{\in }}{\mathcal{A}}}{d}_{i,j,t}$$

The constraints are presented below. They are divided into four different groups (routing, energy, charging, and battery degradation costs) to facilitate the understanding of the formulation.

### Routing constraints

Constraints (5–10) relate to the fleet’s transportation service, covering riding, reallocation, and parking periods.

5 $${d}_{i,j,t+1}={d}_{i,j,t}+{P}_{i,j,t}-\mathop{\sum }\limits_{k{\mathscr{\in }}{\mathcal{K}}}{x}_{k,i,j,t}\,,\,\forall \left(i,j\right){\mathscr{\in }}{\mathcal{A}},\forall t\in {\mathcal{T}}$$

6 $${z}_{k,i,t}=0,k{\mathscr{\in }}{\mathcal{K}},i{\mathscr{\ne }}{\mathcal{S}}{\mathscr{\cup }}{\mathscr{O}},\forall t\in {\mathcal{T}}$$

7 $${p}_{k,i,t}=\mathop{\sum }\limits_{\left(j,i\right){\mathscr{\in }}{\mathcal{A}}:{\tau }_{j,i}\le t}\left({x}_{k,j,i,t-{\tau }_{j,i}}+{y}_{k,j,i,t-{\tau }_{j,i}}\right),\,\forall k{\mathscr{\in }}{\mathcal{K}},\forall i{\mathscr{\in }}{\mathscr{N}},\forall t{\mathscr{\in }}{\mathcal{T}}$$

8 $$\mathop{\sum }\limits_{i{\mathscr{\in }}{\mathscr{N}}}\left({z}_{k,i,t}+\mathop{\sum }\limits_{\left(i,j\right){\mathscr{\in }}{\mathcal{A}}}\left({x}_{k,i,j,t}+{y}_{k,i,j,t}\right)\right)\le 1,\forall k{\mathscr{\in }}{\mathcal{K}},\forall t{\mathscr{\in }}{\mathcal{T}}$$

9 $${z}_{k,i,t}+{p}_{k,i,t}\le 1,\forall k{\mathscr{\in }}{\mathcal{K}},\,i{\mathscr{\in }}{\mathscr{N}},t{\mathscr{\in }}{\mathcal{T}}$$

10 $${z}_{k,i,t+1}={z}_{k,i,t}+{p}_{k,i,t}-\mathop{\sum }\limits_{\left(i,j\right){\mathscr{\in }}{\mathcal{A}}}\left({x}_{k,i,j,t}+{y}_{k,i,j,t}\right),\forall k{\mathscr{\in }}{\mathcal{K}},\,\forall i{\mathscr{\in }}{\mathscr{N}},\forall t{\mathscr{\in }}{\mathcal{T}}$$

Constraint (5) tracks the number of passengers waiting (${d}_{i,j,t+1}$) in node $i$ to travel to $j$ at timestep $t+1$. It is the sum of the number of the passengers already waiting (${d}_{i,j,t}$) plus the new passenger arrivals (${P}_{i,j,t}$), minus the current vehicle demand served (${x}_{k,i,j,t}=1$). Constraint (6) guarantees that the vehicle $k$ is only parked (${z}_{k,i,t}=1$) at a parking spot ($s\epsilon {\mathscr{S}}$) or in an outage location ($o\epsilon {\mathscr{O}}$). Constraint (7) states that a vehicle $k$ has arrived at a node $i\in {\mathscr{N}}$ (i.e., ${p}_{k,i,t}=1$) in the expected travel time ${\tau }_{i,j}$, after carrying a passenger (${x}_{k,i,j,t}=1$) or being reallocated (${y}_{k,i,j,t}=1$). Constraint (8) states that a vehicle $k$ can only be at one of the possible states: parked (${z}_{k,i,t}=1$) or moving (${x}_{k,i,j,t}=1$ or ${y}_{k,i,j,t}=1$). Constraint (9) guarantees the continuity of the parking period, i.e., if a vehicle $k$ is at a node $i$, it must have arrived or being idle. Constraint (10) states that a vehicle $k$ only stays parked at a node $i$ at a timestep $t+1$, if it was already parked or have arrived in the precedent timestep $t$.

### Energy constraints

Constraints (11–13) relate to the vehicles’ energy levels and the battery charging bounds.

11 $${e}_{k,t+1}={e}_{k,t}+\mathop{\sum }\limits_{s{\mathscr{\in }}{\mathcal{S}}}\eta .{g}_{k,s,t}^{{ch}}-\mathop{\sum }\limits_{o{\mathscr{\in }}{\mathscr{O}}}\frac{1}{\eta }.{g}_{k,o,t}^{{out}}-\xi .\left(1-\mathop{\sum }\limits_{i{\mathscr{\in }}{\mathscr{N}}}{z}_{k,i,t}\right),\,\forall k{\mathscr{\in }}{\mathcal{K}},\forall t{\mathscr{\in }}{\mathcal{T}}$$

12 $${\gamma }^{\min }\le {e}_{k,t}/{B}_{k}\le {\gamma }^{\max }\,,\,\forall k{\mathscr{\in }}{\mathcal{K}},\forall t{\mathscr{\in }}{\mathcal{T}}$$

13 $${e}_{k,0}={\gamma }_{k}^{0}.{B}_{k}\,,\forall k{\mathscr{\in }}{\mathcal{K}}$$

Constraint (11) tracks the energy level ${e}_{k,t+1}$ of vehicle $k$ as the energy level ${e}_{k,t}$ at previously timestep $t$, plus the energy charged ${g}_{k,t}^{{ch}}$, minus the energy discharged ${g}_{k,t}^{{out}}$ in a location affected by an outage minus the energy consumed per timestep $\xi$ during a trip (${z}_{k,i,t}=0$). Constraint (12) ensures that the battery of vehicle $k$ with capacity ${B}_{k}$ is never charged above the maximum state of charge (SOC) ${\gamma }^{\max }$ or below the minimum SOC ${\gamma }^{\min }$. Constraint (13) establishes the initial battery level ${\gamma }_{k}^{0}$ for each vehicle $k$ at $t=0$.

### Charging constraints

Constraints (14–17) relate to charging/discharging events at parking spots and at outage locations.

14 $${g}_{k,s,t}^{{ch}}\,\le {\rho }^{{ch}}.{u}_{k,s,t}^{{ch}}\,,\forall k{\mathscr{\in }}{\mathcal{K}},\forall s{\mathscr{\in }}{\mathscr{S}},\forall t{\mathscr{\in }}{\mathcal{T}}$$

15 $${g}_{k,o,t}^{{out}}\,\le {\rho }^{{out}}.{u}_{k,o,t}^{{out}}\,,\forall k{\mathscr{\in }}{\mathcal{K}},\forall o{\mathscr{\in }}{\mathscr{O}},\forall t{\mathscr{\in }}{\mathcal{T}}$$

16 $${u}_{k,i,t}^{{ch}}+{u}_{k,i,t}^{{out}}\le {z}_{k,i,t+1}\,,\forall k{\mathscr{\in }}{\mathcal{K}},\forall i{\mathscr{\in }}{\mathscr{S}}{\mathscr{\cup }}{\mathscr{O}},\forall t{\mathscr{\in }}{\mathcal{T}}$$

17 $$\mathop{\sum }\limits_{k{\mathscr{\in }}{\mathcal{K}}}{g}_{k,o,t}^{{out}}\le {E}_{o,t}^{{req}},\forall o{\mathscr{\in }}{\mathscr{O}},\forall t{\mathscr{\in }}{\mathcal{T}}$$

Constraints (14–15) define the energy charged and discharged of the vehicles at parking spots or outage locations. Constraint (16) guarantees that a vehicle $k$ can only perform one of these actions if parked (${z}_{k,i,t+1}=1$) at each timestep t. Constraint (17) states that the energy provided by the vehicles is not above the energy required ${E}_{o,t}^{{req}}$ in an affected region.

### Battery degradation constraints

Constraints (18–19) evaluate the degradation costs incurred by providing energy to the grid, using an Ah-throughput approach^30,31^. This method estimates degradation costs from charging by accounting for the total energy the battery can cycle through to the end of its life.

18 $${a}_{k}={N}^{{Cy}}.{\lambda }^{{DoD}}.{B}_{k},\forall k{\mathscr{\in }}{\mathcal{K}}$$

19 $${\omega }_{k,t}=\left(\frac{{R}^{{bat}}.{B}_{k}}{{a}_{k}}\right).\mathop{\sum }\limits_{i{\mathscr{\in }}{\mathscr{N}}}\left({g}_{k,i,t}^{{out}}\right),\forall k{\mathscr{\in }}{\mathcal{K}},\forall t{\mathscr{\in }}{\mathcal{T}}$$

Constraint (18) calculates the total energy ${a}_{k}$ drawn from a vehicle $k$ battery throughout its lifespan, considering the total number of cycles ${N}^{{Cy}}$, depth of discharge ${\lambda }^{{DoD}}$, and total battery capacity ${B}_{k}$. Constraint (19) defines the costs ${\omega }_{k,t}$ related to the battery degradation of a vehicle $k$ due to outage discharging events. The first term (${R}^{{bat}}.{B}_{k}/{a}_{k}$) represents a conversion factor that translates the total energy throughput into a monetary value. In contrast, the second one ($\mathop{\sum }\limits_{i\in {\mathscr{N}}}\left({g}_{k,i,t}^{{out}}\right)$) assesses the amount of energy charged within a specific timestep.

### Reduce-form restauration feedback

We endogenize the residual deficit to capture the first-order coupling between SAEV supply and restoration dynamics. The discharge is presented in Eq. (20):

20 $$\mathop{\sum }\limits_{k{\mathscr{\in }}{\mathcal{K}}}\mathop{\sum }\limits_{o{\mathscr{\in }}{\mathcal{O}}}{g}_{k,o,t}^{{out}}={E}_{t}^{{SAEV}},\forall t{\mathscr{\in }}{\mathcal{T}}$$

With a critical share of energy provided to high-priority zones and the remainder to non-critical loads, defined in Eqs. (21–22):

21 $${E}_{t}^{{crit}}={E}_{t}^{{SAEV}}.{\psi }_{t}^{{crit}},\forall t{\mathscr{\in }}{\mathcal{T}}$$

22 $${E}_{t}^{{out}}=\left({E}_{t}^{{SAEV}}-{E}_{t}^{{crit}}\right),\forall t{\mathscr{\in }}{\mathcal{T}}$$

where ${\psi }_{t}^{{crit}}$ is a percentage of the total energy is provided to critical zones at each timestep. Then, the next-step residual deficit updates as in Eq. (23):

23 $${D}_{t}=\max \left\{0,{D}_{t+1}^{{orig}}-\beta .{E}_{t}^{{out}}-\alpha .{E}_{t}^{{crit}}\right\}$$

where ${D}_{t}^{{orig}}$ is the total energy required at timestep t, ${D}_{t}$ is the new deficit after the SAEV discharging, $\beta$ is the system-wide relief elasticity for non-critical discharge and $\alpha$ the incremental efficacy when discharge targets critical nodes ($\alpha ,\beta \in [\mathrm{0,1}]$). The max operator enforces non-negativity. We add this reduced-form endogeneity because an exogenous deficit path treats SAEV discharge as orthogonal to restoration; the linear update in Eq. (22) captures the first-order elasticity of restoration with respect to distributed energy and the incremental efficacy of critical targeting, while preserving MILP tractability.

### Solution algorithm

In this study, we utilize the ε-constraint method to calculate the non-dominated (Pareto) front of the MOO problem^32^. This method has distinct advantages over other solution approaches. Unlike heuristic methods, which often produce suboptimal solutions, this algorithm enables the use of exact optimization techniques to identify optimal solutions with off-the-shelf solvers. Furthermore, it does not require arbitrary value weights and can recover unsupported efficient solutions that weighted sums or heuristics might overlook. This choice also has a direct policy interpretation, as the secondary objective acts as a service guardrail, with each point representing a guaranteed minimum service level. Lastly, as a well-established method with straightforward implementation, it provides a robust and reliable framework for addressing the inherent trade-offs in MOO problems.

We keep the profit function (${f}_{1}$) as the objective to be optimized and convert the riding service function (${f}_{2}$) into a constraint. A bound ${\varepsilon }_{n}$ is imposed for the new constraint at each iteration $n$. The complete optimization problem is defined as follows:

24 $$\max {f}_{1}=\mathop{\sum }\limits_{t{\mathscr{\in }}{\mathcal{T}}}\left({f}_{t}^{{trip}}-{f}_{t}^{{eng}}\right)$$

s.t.

25 $$\mathop{\sum }\limits_{t{\mathscr{\in }}{\mathcal{T}}}\mathop{\sum }\limits_{\left(i,j\right){\mathscr{\in }}{\mathcal{A}}}{d}_{i,j,t}\le {\varepsilon }_{n},n=2,\ldots ,\,N$$

Constraints (5)-(19).

To avoid starting the iteration with a weakly non-dominated solution, we obtain ${\varepsilon }_{1}$ by computing the optimal value in (24), where $\delta$ is set as 0.0001. This lexicographic perturbation avoids a weakly non-dominated starting point.

26 $$\max ({f}_{1}-{\delta .f}_{2})$$

s.t.

Constraints (5–19).

We begin by determining ε₁ via an optimization of the modified objective ${f}_{1}-{\delta .f}_{2}$ (with $\delta$ ε = 0.0001), ensuring a feasible initial solution that is not weakly dominated. Subsequently, we reduce ε by a fixed step (σ = 250) in each iteration (*N* = 18). This systematic tightening of the ${f}_{2}$ constraint enables us to explore the trade-offs between profit (${f}_{1}$) and the riding service function (${f}_{2}$), ultimately constructing a Pareto front of non-dominated solutions using off-the-shelf solvers. Finally, we present the proposed ε-constraint method in Algorithm 1.

#### Algorithm 1

ε-constraint method
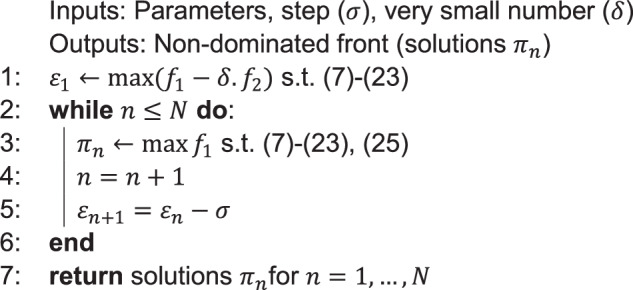


### Case study

**Fig. 4:**
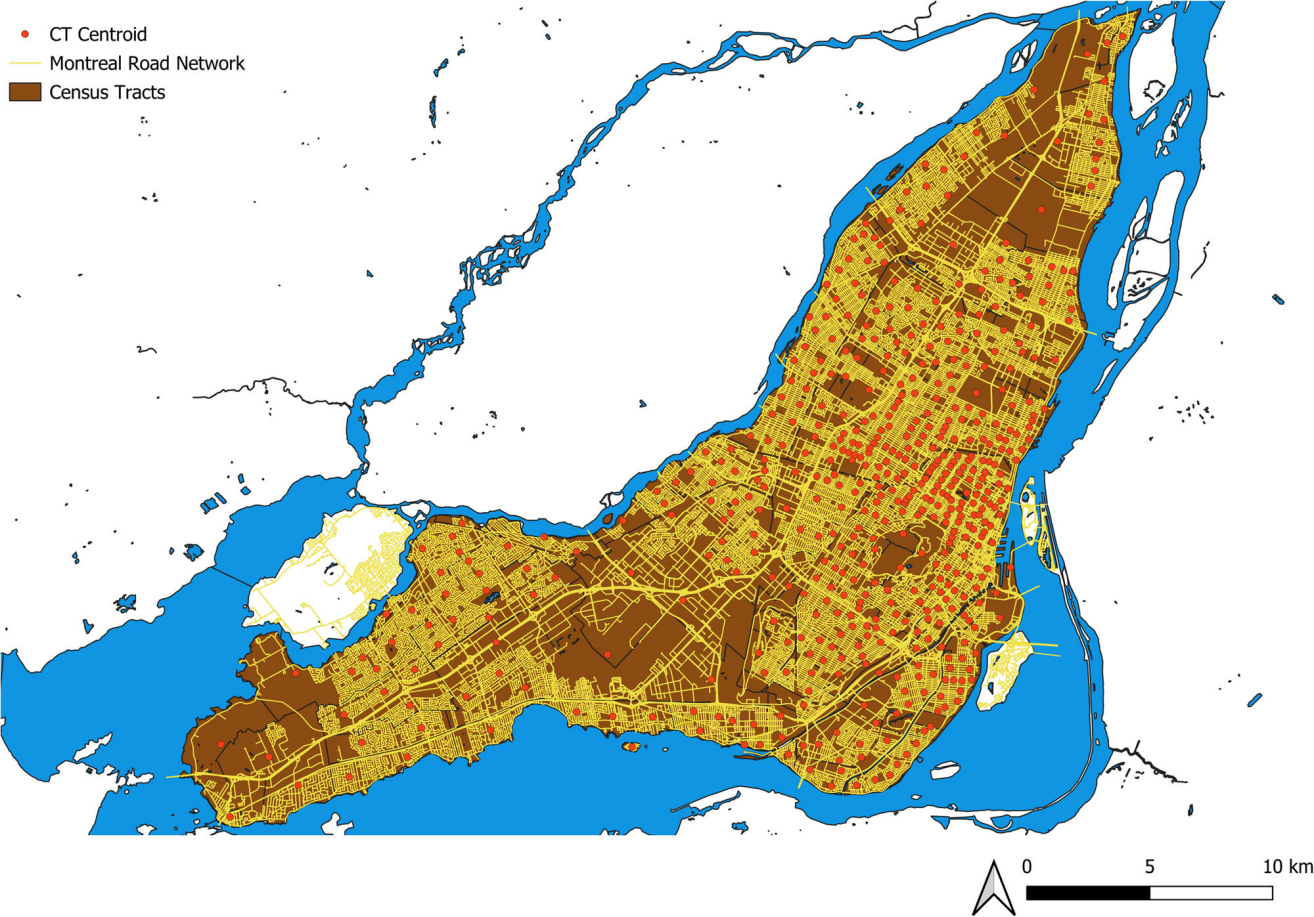
Montreal’s area evaluated in this paper.

Montreal (Quebec, Canada) provides a relevant testbed, given its dense urban form and exposure to weather-related disruptions (e.g., snowstorms, heatwaves), as well as open data availability. Figure 4 shows the study area, including the road network and zone centroids used for modeling.

We simulate a hypothetical SAEV system that both serves trips and supports outage mitigation. For tractability, pick-ups and drop-offs occur at zone centroids; this abstraction reduces computational load while preserving routing realism for city-scale analysis.

### Origin-destination demand

Travel demand is obtained from the Montreal OD survey^33^, which samples ~5% of residents and supplies expansion factors to recover population totals. Origins and destinations are anonymized to a 250 m grid. For this study, we retain only trips with both origin and destination on the island of Montreal.

**Fig. 5:**
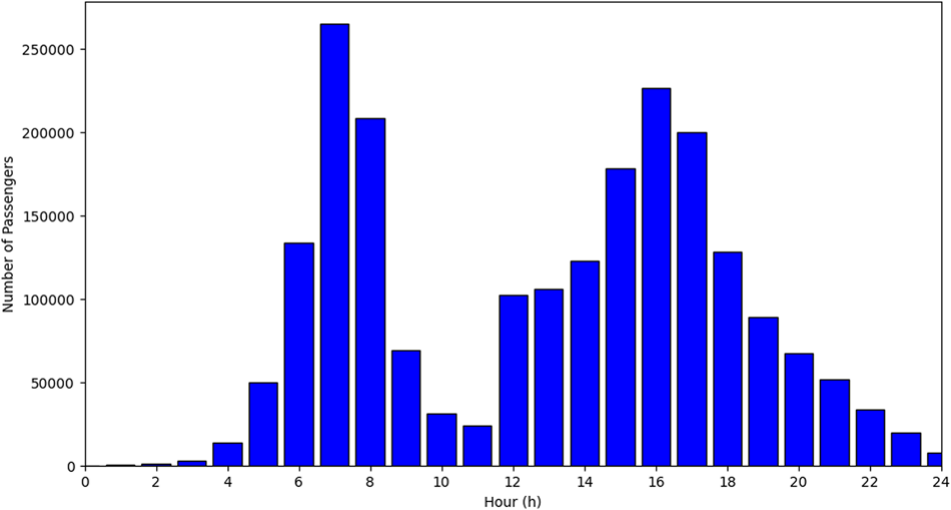
Origin-destination demand evaluated in this paper.

The island is partitioned into 526 Statistics Canada census tracts (CTs)^34^ (an average area of 0.89 km²). We construct hourly OD matrices by aggregating expansion factors to the CT–CT cells according to each trip’s departure hour. Network distances are computed as shortest-path lengths on Montreal’s open-access road network between CT centroids that are snapped to the network (QGIS)^35^. To reduce computation, only the lower triangular of the distance matrix is evaluated and mirrored; any asymmetry from one-way links is negligible given a mean centroid–centroid shortest path exceeding 12.5 km. Figure 5 presents the island-only subset used in the optimization, exhibiting the expected morning and evening peaks.

### SAEV fleet characteristics

We model a medium-sized fleet of 100 SAEVs to strike a balance between realism and computational tractability for a city of Montreal’s scale. Vehicles are equipped with 80 kWh batteries, consistent with current mass-market EVs, and feature a conservative, neighborhood-compatible charging infrastructure: 10 kW charging and 7.2 kW bidirectional discharging. This choice prioritizes near-term deployability (lower hardware/interconnection costs; compatibility with Type 1/2 AC) and limits peak stress on local feeders relative to high-power DC systems. Table 3 outlines the key parameters considered in the SAEV system. We explore the economic implications of three distinct tariffs for the two service types. Firstly, a charging tariff of 0.10 CAD$/kWh applies when vehicles recharge at designated stations across the city. Secondly, a discharging tariff of 0.45 CAD$/kWh is applied when SAEVs are deployed to provide support during power outage events. Additionally, a riding services tariff of 0.50 CAD$ per trip is implemented to cover the cost of passenger transport using SAEV services.

**Table 3:** Parameters used in the baseline case

Parameter	Value
Fleet size	100
Battery capacity	80 kWh
Energy consumption	0.2 kWh/km
Average speed	30 km/h
Charging power	10 kW
Discharging power	7.2 kW
Charger efficiency	90%
Battery replacement costs	150 CAD$/kWh^36^
Number of cycles	3000^37^
Depth of discharge	60%

### Power outage events

**Fig. 6:**
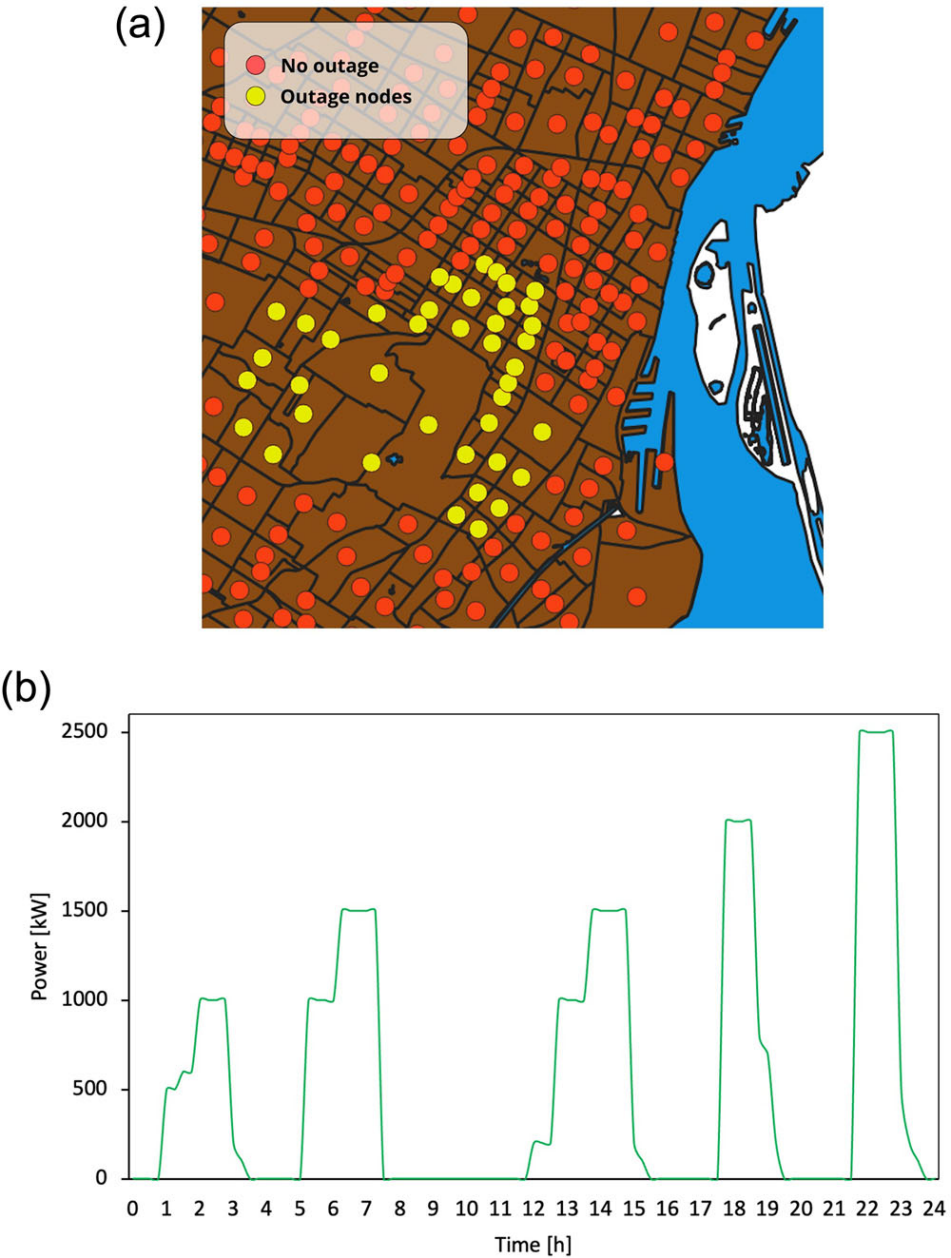
Outage representation: **a** affected nodes in the downtown footprint, and **b** residual power deficit used in the scenarios, constructed from Hydro-Québec outage statistics with staged restoration and local backup embedded.

We focus on the operational response of an SAEV fleet during power outage events. The electric grid is represented exogenously by a time-varying residual outage profile within the study footprint, which already reflects staged restoration and local backup. The optimizer, therefore, reacts to the net shortfall that remains to be served. Figure 6 illustrates the total power demand in the affected zones as the baseline under a disruption.

We model power outage scenarios to test a range of severities across the day within the downtown footprint. Figure 6a identifies the affected nodes, and Fig. 6b shows the time profile of the residual power deficit used in the simulations. The profile is derived from Hydro-Québec monitoring data (https://infopannes.solutions.hydroquebec.com/info-pannes) on outage scale, frequency, and duration by placing several pulses of 60–150 min and scaling their peaks to 1.0–2.4 MW to reflect staged restoration and local backup. For context, a 2.4 MW deficit corresponds to roughly 1600–2400 households, assuming coincident power demand of 1.0–1.5 kW per household in the affected area. Pulses occur during both low- and high-mobility periods, thereby stressing the fleet under contrasting operating conditions. Larger regional power outages, which can involve up to 10,000 households, fall outside the modelled footprint and would require proportionally larger fleets, as noted in the sensitivity and policy discussions.

## Results

In this section, we present the results of our experiments. The model is implemented in Python and solved using Gurobi with a 15-min timestep. We chose this timestep discretization to balance computational efficiency and tractable results. If a travel time does not align with 15 min, it can be rounded to a multiple of this interval. All experiments were run on Linux (Kubuntu 18.04) with an Intel Xeon Gold 6138 processor (80 cores, 2.0 GHz) and 314 GB of RAM.

### Non-dominated front

**Fig. 7:**
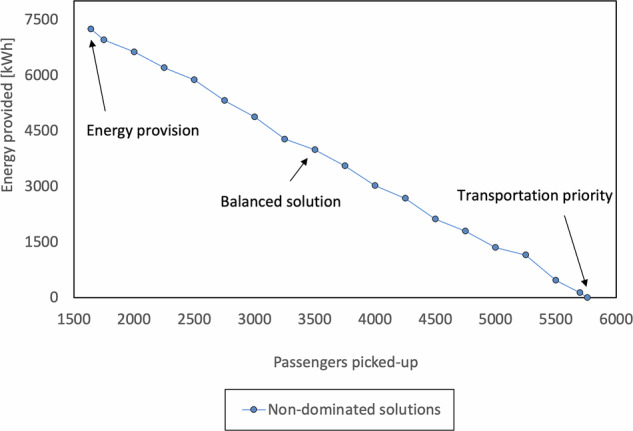
Pareto front obtained through the optimization procedure (highlighted are the extreme and balanced solutions).

Figure 7 presents the Pareto front for the developed MOO model, showing the values that individually optimize each objective function (extreme solutions) and, in between, solutions that represent various trade-offs around the different objectives.

From the obtained front, we highlight three key scenarios from our analysis: a balanced solution, and two extremes labeled “Transportation only” and “Energy provision.” The Transportation only case dedicates the entire fleet to passenger transport, resulting in zero energy delivery and 5692 passenger pick-ups. Conversely, the Energy provision case prioritizes grid support, delivering 7238 kWh of energy (roughly meeting the daily energy demand of 180 houses), while servicing only 1556 passenger pick-ups. The balanced scenario serves as our benchmark, achieving moderate values for energy delivery (3986 kWh) and passenger transport (3556 pick-ups), thereby offering a compromise between the two objectives. These comparisons underscore the importance of a balanced approach in managing the trade-off between energy provision and passenger services, with the optimal solution ultimately depending on the SAEV-O’s specific priorities and constraints. Table 4 provides a detailed comparison of these three scenarios.

**Table 4:** Comparison of the three highlighted solutions

	Balanced	Transportation priority^*^	Energy provision^*^
Energy delivered [kWh]	3986	0	7238 (+81.58%)
Energy charged [kWh]	9105.75	8772.75 (−4%)	9882 (+8.5%)
Passenger pick-ups	3556	5692 (+38%)	1556 (−56%)
Total revenues [CAD$]	2657.80	1968.72 (−35%)	3040.87 (+14%)

*The values in parentheses indicate the variation between the extreme and balanced scenarios.

### Extreme cases

To deepen the analysis, we comprehensively evaluate the two extreme cases: (a) Transportation priority and (b) Energy provision.

### Transportation priority

**Fig. 8:**
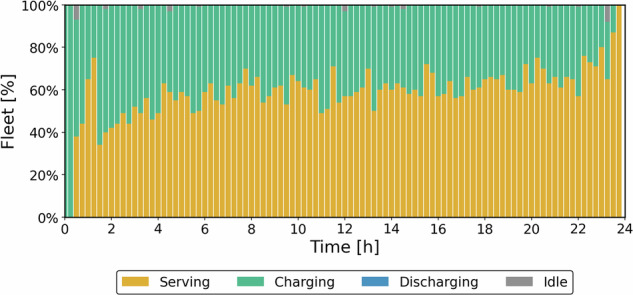
Status of the fleet during operation (Transportation priority case).

Figure 8 illustrates the state of each SAEV throughout a day, when the entire fleet is engaged in transportation services.

**Fig. 9:**
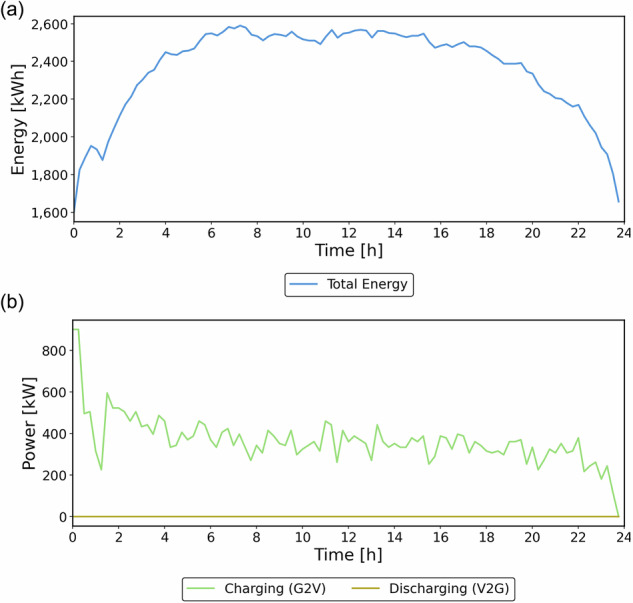
Energy and power curves: **a** Energy level and **b** Charging power (Transportation priority case).

The optimization schedules coordinated charging to limit simultaneous sessions, reducing charger congestion, maintaining fleet availability, and decreasing passenger wait times. Across the horizon, about 60–65% of vehicles are in service at any time, and the fleet serves 5692 passengers. During operation, the model maintains a minimal number of vehicles on idle to minimize energy consumption, avoid unnecessary charging, and prevent unprofitable trips. Figure 9 presents the total energy level and charging events during operation. Discharge events are zero because there are no SAEVs allocated during an outage.

Vehicles start the day with approximately 20% state of charge (SOC), reflecting the depletion that occurred at the end of the previous day. A peak occurs around 00:00–1:00 when demand is low, and vehicle charge levels are at their lowest after evening use; afterwards, SOC decreases gradually as trips increase. The optimization maintains SOC within safe limits, reducing degradation, with a significant drawdown only occurring late in the day when charging options are limited.

### Energy provision

**Fig. 10:**
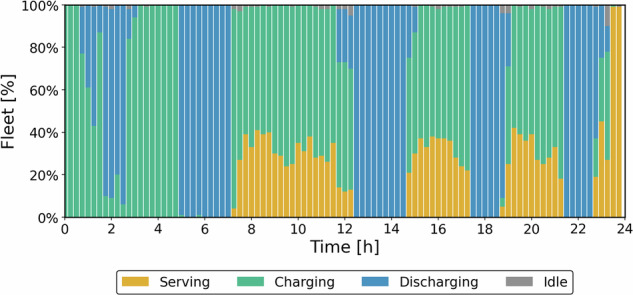
Status of the fleet during operation (Energy provision case).

Figure 10 shows the status of the vehicles in the energy provision case throughout the operational day. The optimized results yield 3,040.87 CAD$ in revenue, ~54% above the transportation-priority case, because the discharge tariff is set sufficiently above the purchase price to make energy arbitrage profitable.

**Fig. 11:**
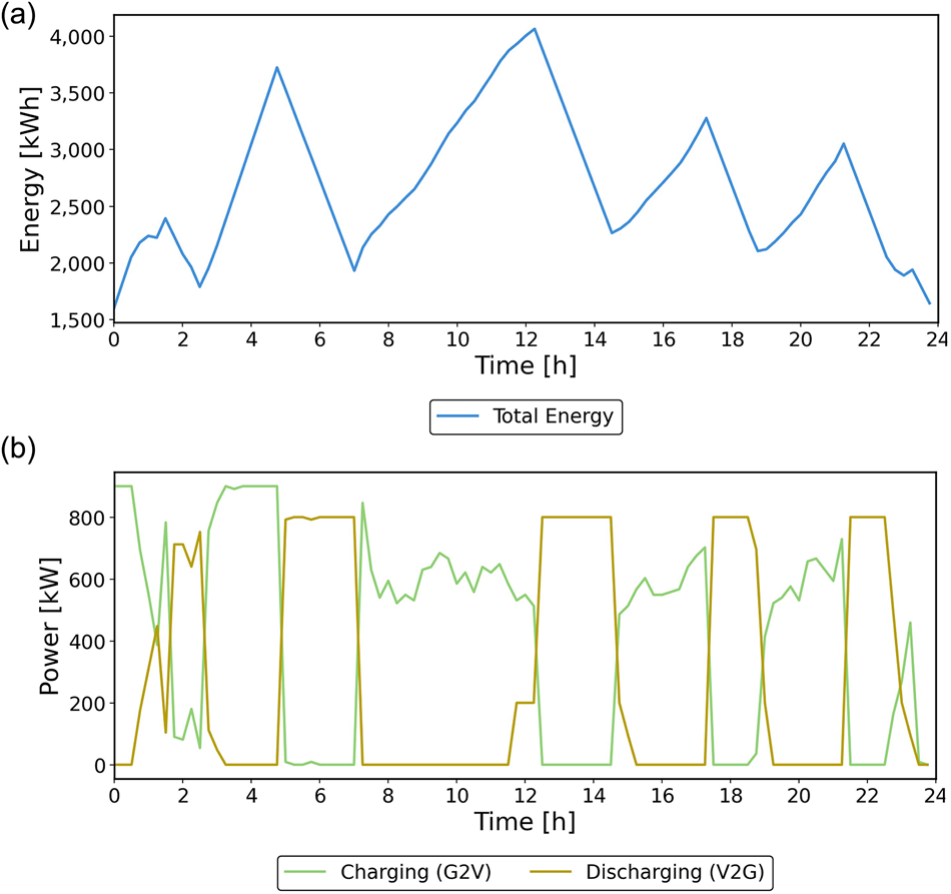
**Energy and power curves: a** Energy level and **b** charging power (Energy provision case).

In the energy priority case, vehicles spend most of their time charging and discharging, and they serve rides mainly when no outage is active. This is not ideal, since the fleet’s primary role is mobility, but it makes the trade-off clear. To supply energy during outages, the fleet must first build and maintain an SOC buffer and travel to affected areas, which reduces the time available for trips. Charging therefore brackets discharge periods so there is enough SOC for delivery and safe operation. Figure 11 presents the total energy level and discharging/charging events during operation.

**Fig. 12:**
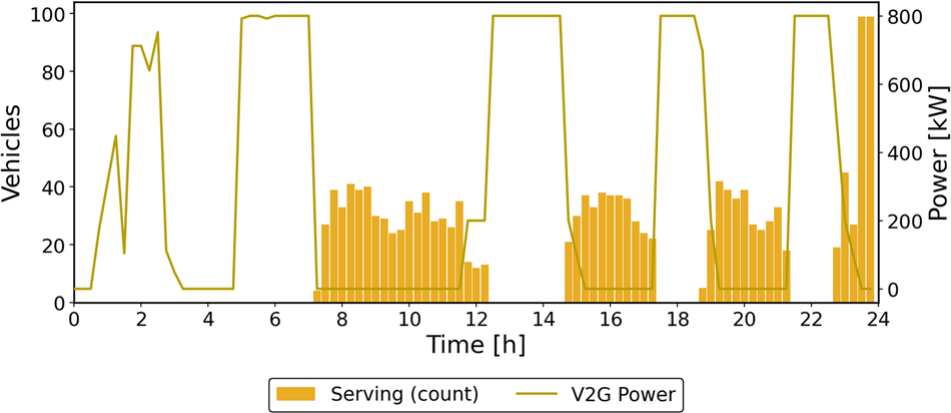
Number of vehicles serving (%) and passenger demand (Energy provision case).

Peak charging occurs during early off-peak hours, when many vehicles are plugged in before passenger and outage activity begins. Once a target SOC is reached, part of the fleet discharges at the affected nodes while the rest continues charging, creating a sawtooth energy profile from alternating charge–discharge cycles. These cycling speeds up battery degradation; however, the model incorporates a degradation cost, so discharge is scheduled only when it remains profitable for the operator. Figure 12 compares the percentage of vehicles in service with the passenger demand at each time interval.

**Fig. 13:**
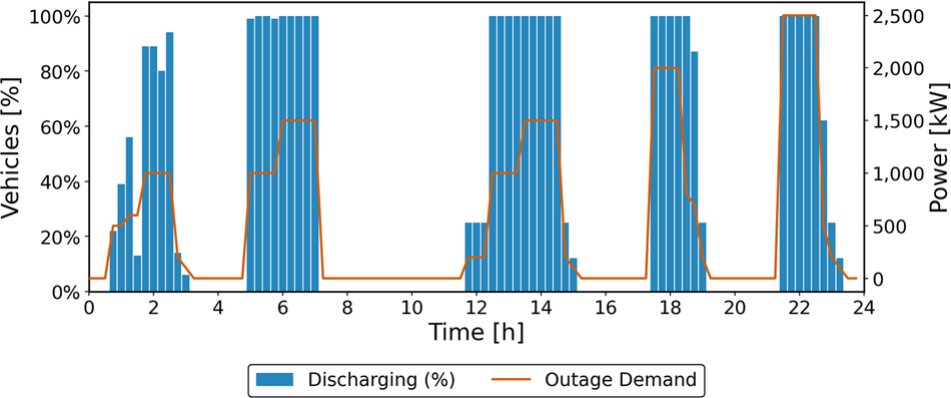
Number of vehicles offering energy to the grid (%) and power demand (Energy provision case).

The vehicles serve passengers only when no outage event is occurring. Consequently, only 1556 passengers are transported, representing a 72.6% decrease compared to the transportation priority scenario. Figure 13 contrasts the percentage of vehicles selling energy with the power demand at each timestep.

The results of this scenario indicate that the fleet can deliver 7238 kWh during the analyzed period, accounting for approximately 28% of the total energy needs. This finding suggests that SAEV fleets could function as “emergency” storage systems, supplying energy to and from various points within a city. However, this dual role must be carefully managed to avoid jeopardizing the SAEV-O’s primary purpose of transporting passengers. Note that the analysis focused on relatively minor outage events typical for a city like Montreal. In cases of more severe outages, the energy requirements would likely exceed the fleet’s storage capabilities, as outlined in this example. To address such scenarios, a larger fleet would be necessary to mitigate grid issues effectively. While alternatives such as generators or stationary batteries are available, their high upfront and operating costs often make them impractical for unexpected outages. Considering the limited viable alternatives, integrating SAEVs as mobile energy storage presents a promising strategy to enhance urban resilience. In this context, our analysis suggests that without an attractive remuneration scheme, offering energy to the grid can significantly impact the profits of SAEV-Os, making them less likely to provide such services during outages.

### Feedback impact on restauration

**Fig. 14:**
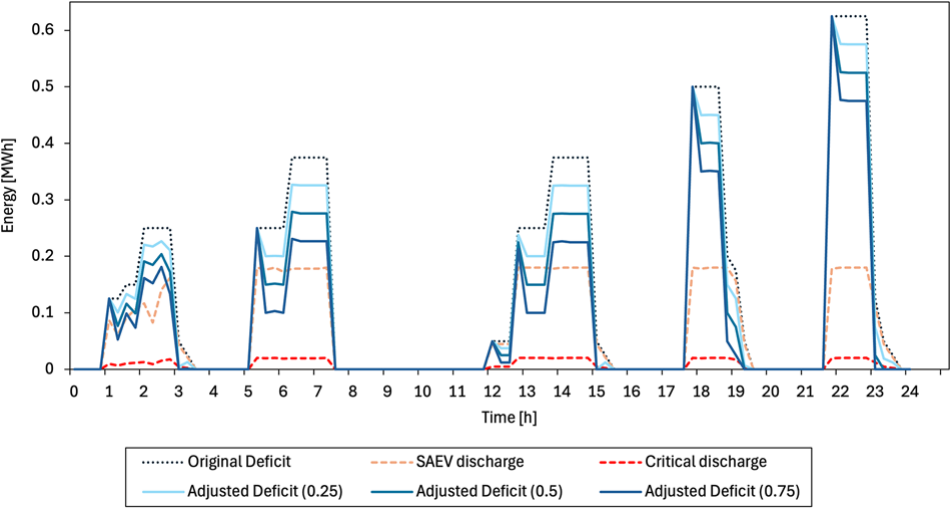
Deficit trajectories under one-step restoration feedback.

We evaluate the one-step feedback (see Eq. 22) with fixed dispatch and $(\beta ,\alpha )\in \{\left(\mathrm{0.25,0.25}\right),\left(\mathrm{0.50,0.50}\right),\left(\mathrm{0.75,0.75}\right)\}$. Figure 14 contrasts the exogenous envelope ${D}_{t}^{\mathrm{orig}}$ with the adjusted trajectories ${D}_{t}$.

The results indicate a decrease in residual deficit when SAEVs discharge, while the envelope remains unchanged in other cases. Across all bursts, peaks become narrower and tails shorten; full restoration occurs 15–45 min earlier, with larger improvements as $(\beta ,\alpha )$ increase. These effects are mainly seen within energy-limited windows; outside these windows, the trajectories are similar.

Furthermore, we measure the impact of the one-step feedback using two metrics: (i) energy not served (ENS) and (ii) recovery time. ENS aggregates the residual deficit at 15-min resolution. For scenario $X$, when the deficit is expressed as energy per 15-min step (MWh), ENS is defined in Eq. (27):

27 $${{ENS}}^{(X)}=\mathop{\sum }\limits_{t=1}^{T}{D}_{t}^{(X)}$$

Recovery time captures dynamics over each baseline-identified burst. Let $[{\tau }_{b},{e}_{b}]$ be the baseline burst window, ${\tau }_{b}$ its peak time, and $\Delta t$ the analyzed timestep. Using a common threshold equal to five percent of the baseline peak $0.05{D}_{{\tau }_{b}}^{(\mathrm{base})}$, the recovery time for scenario $X$ is given by Eq. (28):

28 $${T}_{\mathrm{rec}}^{(X,b)}=(\min \{t\in [{\tau }_{b},{e}_{b}]:{D}_{t}^{(X)}\le 0.05\,{D}_{{\tau }_{b}}^{(\mathrm{base})}\}-{\tau }_{b})\Delta t$$

We used the common baseline scenario for like-for-like comparisons. In this setting, a lower ENS indicates less unmet energy, and a shorter recovery time signifies quicker stabilization. Table 5 presents the metrics results.

**Table 5:** Outage restauration metrics

Scenario	ENS (MWh)	Avg. recovery time to 5% (min)
Baseline (No feedback)	13.35	84.0
β = α = 0.25	11.62 (−12.9%)	75.0 (−10.7%)
β = α = 0.50	10.03 (−24.9%)	72.0 (−14.3%)
β = α = 0.75	8.53 (−36.1%)	69.0 (−17.9%)

### Sensitivity analysis

To further evaluate the results, we conduct a sensitivity analysis by varying different parameters of the default case study. Table 6 presents the main characteristics of the different evaluated scenarios. Except for the parameter under assessment, the remaining parameters in each scenario adhere to the previously stated nominal values. The results will be presented considering the transportation priority and energy provision cases previously described.

**Table 6:** Analyzed scenarios

Scenario	Description	Deviation
S1.1, S1.2	Variation in the number of SAEVs available.	80, 120 vehicles
S2.1, S2.2	Variation in the battery capacity of the fleet.	60, 100 [kWh]
S3.1, S3.2	Variation in charging power.	5, 15 [kW]
S4.1, S4.2	Variation in discharging power.	5, 15 [kW]
S5.1, S5.2	Variation in discharging tariff	0.25, 0.6 [CAD$/kWh]

### Transportation priority

Table 7 presents the results for the transportation priority case. Note that scenarios S4 and S5 are not evaluated because they involve discharging activities, which are not part of this configuration.

**Table 7:** Sensitivity analysis results (Transportation priority case)

Scenario	Revenues [CAD$]	Number of passengers	Energy charged [kWh]
Base	1968.72	5692	8772.75
S1.1	1636 (−20.3%)	4640 (−22.7%)	6840 (−28.3%)
S1.2	2035.95 (+3.3%)	5781 (+1.5%)	8545.5 (−2.7%)
S2.1	2026.225 (+2.8%)	5762 (+1.2%)	8547.75 (−2.6%)
S2.2	2036.95 (+3.3%)	5783 (+1.6%)	8545.5 (−2.7%)
S3.1	1441.95 (−36.5%)	4098 (−38.9%)	6070.5 (−44.5%)
S3.2	2316.9 (+15%)	6594 (+13.7%)	9801 (+10.5%)

**Fig. 15:**
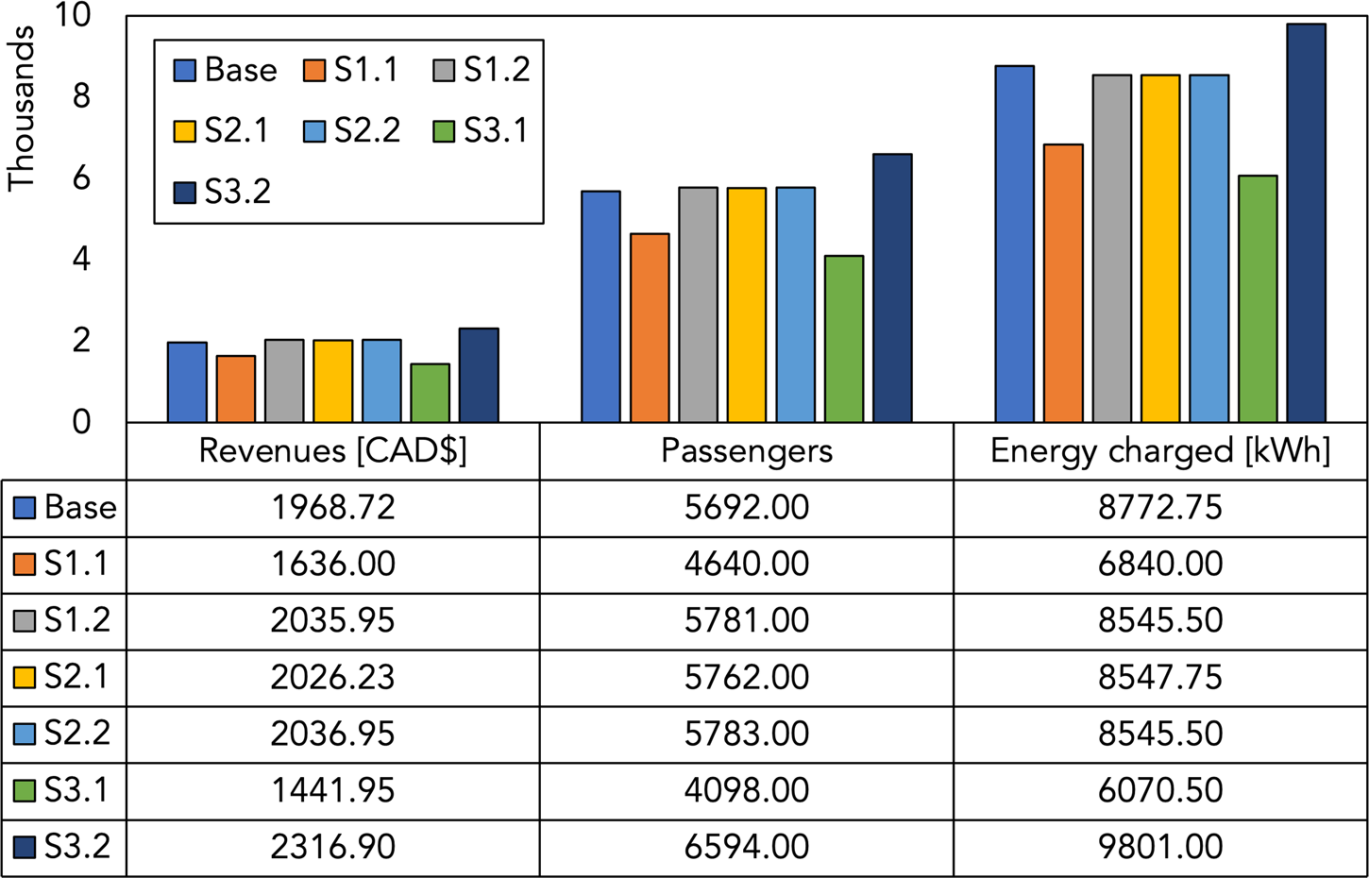
Variation of revenues, number of passengers, and energy charged (Transportation priority case).

To better visualize the results and the impact of changing the parameters, Fig. 15 is introduced.

The analysis highlights the effects of varying operational parameters on revenues, passenger transportation, and energy usage within the transportation priority scenario. The main implications are introduced in the following:

- **Revenues:** The base case shows revenues of CAD$ 1968.72. With a 20% reduction in fleet size (S1.1), revenues drop by 20.3% (to CAD$ 1636), while a 20% fleet increase (S1.2) only results in a modest 3.3% uplift (to CAD 2035.95). Similarly, changes in battery capacity (S2.1 and S2.2) result in only marginal revenue improvements (~2.8–3.3%). However, enhancing charging power (S3.2) leads to a significant 15% revenue increase (to CAD$ 2316.9), whereas reducing charging power (S3.1) causes a sharp 36.5% decline (to CAD$ 1441.95).
- **Number of passengers:** The base case serves 5,692 passengers. A 20% reduction in fleet size (S1.1) causes a 22.7% drop (to 4640 passengers), while a 20% increase in fleet size (S1.2) results in only a 1.5% rise (to 5781 passengers). Battery capacity variations again yield only slight improvements (~1.2–1.6%), but increasing charging power (S3.2) boosts the number of passengers by 13.7% (to 6594 passengers), whereas lowering charging power (S3.1) reduces the passenger count by 38.9% (to 4098 passengers).
- **Energy charged:** In the base case, the system charges 8772.75 kWh. Under a reduced fleet scenario (S1.1), energy charged drops by 28.3% (to 6840 kWh), and a larger fleet (S1.2) sees a slight reduction of 2.7% (to 8545.5 kWh). Battery capacity changes also yield similar slight reductions (around 2.6–2.7%). Most notably, slower charging (S3.1) results in a 44.5% decrease (to 6070.5 kWh), while faster charging (S3.2) increases energy charged by 10.5% (to 9801 kWh).

The results illustrate a saturation effect as the number of vehicles increases. Although the system currently meets only about 2% of the total passenger demand, its performance is limited by several operational constraints. When the fleet size is increased by 20%, the additional vehicles do not lead to a proportional increase in passengers because other factors, such as charging power, battery capacity, and operational time, become the binding constraints. Essentially, adding more vehicles without addressing these bottlenecks only yields marginal improvements in service. In contrast, reducing the fleet by 20% exacerbates these constraints, leading to a more pronounced decline in passengers served (a 22.7% drop). This asymmetry highlights that the system’s performance is already operating close to its capacity limits imposed by the current charging and operational infrastructure. The analysis shows that improving charging power (as in S3.2) has a significant positive impact. Faster charging allows vehicles to return to service more quickly, increasing the number of trips they can complete and enhancing overall system efficiency. This indicates that investments in high-powered charging infrastructure could be a more effective strategy for increasing revenues and passenger throughput than merely expanding the fleet. In summary, the saturation effect observed with fleet expansion arises because the system is constrained by multiple factors beyond the number of vehicles. While fleet size plays a role, improving the charging infrastructure is the key lever to unlock higher operational performance and better meet passenger demand.

### Energy provision

Table 8 presents the results for the energy priority case, including scenarios S4 and S5, as well as the estimated battery degradation costs.

**Table 8:** Sensitivity analysis results (Energy provision case)

Sc.	Revenues [CAD$]	Number of passengers	Energy provided [kWh]	Energy charged [kWh]	Degradation costs [CAD$]
Base	3040.9	1556	7238	9882	6.03
S1.1	2508.7	1245	5910	7683.75	4.93
S1.2	3,719.72	2091	8524	11,544.75	7.1
S2.1	3119.61	1655	7246	9,625.5	6.04
S2.2	3121.19	1664	7244	9,645.75	6.04
S3.1	2359.45	298	6424	6750	5.35
S3.2	3496.27	2516	7462	11,134.13	6.22
S4.1	2586.08	2021	5359.72	8,318.25	4.47
S4.2	4328.37	1142	11,225	12,845.25	9.35
S5.1	1968.72	5692	0	8772.75	0
S5.2	4197.39	1627	7244	9564.75	6.04

In general, degradation costs are not significantly higher than total revenues, indicating that, from a financial perspective, selling energy to the grid compensates for the degradation incurred from multiple charging events. For scenario S5.1, the remuneration for selling energy to the grid is too low (0.25 CAD/kWh), making these transactions unprofitable. Thus, this scenario adheres to the values of the transportation priority baseline case.

**Fig. 16:**
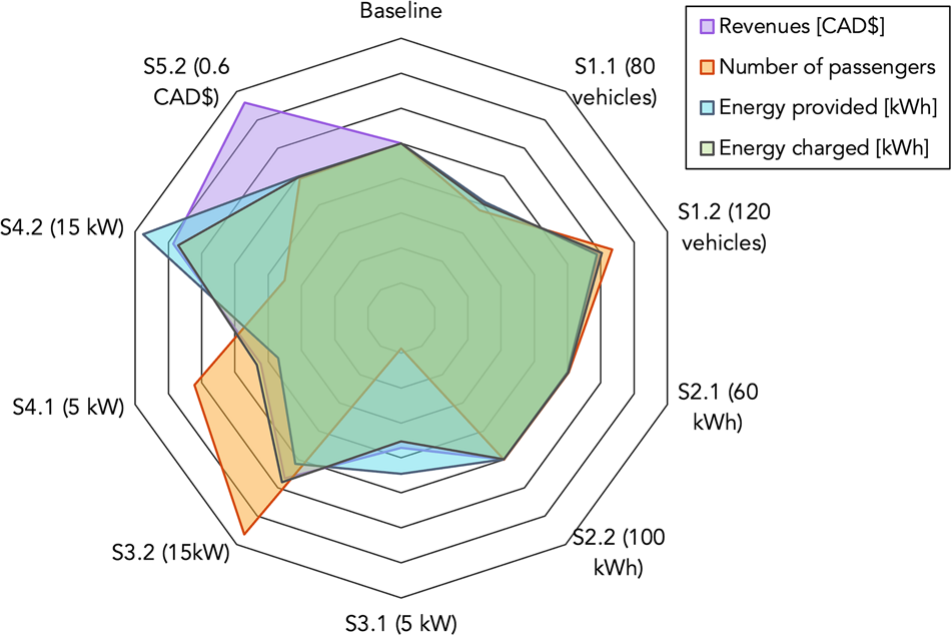
Radar plot (Energy provision case).

Figure 16 presents a radar plot of the results normalized to the baseline scenario. We do not include the results of S5.1 or the degradation costs (which have a curve similar to the “energy provided” one, as these costs are related to the amount of energy sold to the grid).

The results of the sensitivity analysis are described in the following:

- **Revenues:** The analysis indicates that increasing the discharging tariff (S5.2) significantly impacts revenues without substantially affecting the number of passengers transported or the energy charged. This highlights the need for DSOs to offer profitable energy selling margins, making SAEV usage more feasible in the future. Like the transportation priority case, revenues are also positively influenced by increasing the number of vehicles (S1.2) or the charging power (S3.2). However, a larger fleet generates higher revenues due to greater energy flexibility, allowing more energy to be sold back to the grid. Conversely, reducing the number of vehicles and charging power has the opposite effect. Additionally, increasing the discharging power (S4.2) rate increases revenues, enabling the fleet to sell more energy to the grid in a shorter period.
- **Number of passengers:** The analysis shows that increasing the charging power (S3.2) strongly affects the number of passengers carried while still providing energy to the grid. Conversely, reducing the charging power (S3.1) has the opposite effect, as expected, since vehicles spend more time parked for recharging. Additionally, increasing the fleet size (S1.2) positively impacts the number of passengers transported, although not as significantly as increasing the charging power. Interestingly, decreasing the discharging power (S4.1) positively affects the number of passengers carried, as vehicles have less flexibility to be allocated during outage events, leading to more vehicles being engaged in transportation services. On the other hand, increasing the discharging power (S4.2) negatively impacts transportation service levels, as vehicles deplete their batteries faster when delivering more energy to the grid.
- **Energy provided:** Increasing the discharging power (S4.2) enables vehicles to provide more energy to the grid, and at a faster rate. Also, increasing the fleet size (S1.2) has a slight positive effect on the energy provided. However, the limitation of the discharging power hinders the potential of the vehicles in offering energy. This result indicates that, in addition to fleet size, the electricity infrastructure needs to be robust enough to accommodate the potential energy provided by the SAEVs.
- **Energy charged:** The energy charged, i.e., required from the fleet to operate, has major increases in three scenarios (S1.2, S3.2, S4.2). In the cases of S1.2 and S3.2, this increase is related to the possibility of providing more transportation services, as the fleet is larger (S1.2) or can be recharged faster (S3.2). In the case of S4.2, the vehicles can offer more energy to the grid because the discharging facilities are more powerful, meaning that vehicles are often overcharged to provide energy to the grid.

Considering the energy-provision case, the analysis demonstrates that increasing the discharging tariff has a significant impact on revenues, with minimal changes to passenger transportation. This finding highlights the importance of DSOs offering profitable energy selling margins to ensure the long-term viability of SAEVs as auxiliary energy providers. Further, the number of vehicles and the charging power also positively impact revenues, with more powerful chargers having a more pronounced effect due to faster charging times and quicker response capabilities during outages. Moreover, the capacity to provide more energy to the grid is enhanced by increasing discharging power and, to a lesser extent, fleet size. Again, the results indicate that a robust charging infrastructure is crucial for maximizing the energy contribution of SAEVs.

### Thresholds and elasticity analysis

This subsection quantifies decision-relevant thresholds and compares the responsiveness of key performance indicators (KPIs) to policy levers. Table 9 reports the two operational thresholds inferred from the scenarios. The tariff floor defines the price above which V2G participation occurs; the charging-power threshold indicates the minimum power per plug needed to sustain mobility guardrails.

**Table 9:** Thresholds (tariff and power)

Threshold	Range	Operational implication
Tariff floor ($\tau$)	0.25 < $\tau$ ≤ 0.45 CAD/kWh	Below $\tau$ the fleet does not discharge; set event tariffs above $\tau$ to unlock energy support.
Minimum charging power ($\rho$)	5 ≤ $\rho$ ≤ 10 kW	Below $\rho$ mobility degrades sharply; ensure at least $\rho$ before expanding fleet size.

The results show a clear separation of roles. Event prices below the tariff floor do not elicit discharge, whereas prices above it reliably unlock energy support. Likewise, per-plug charging power below the threshold causes sharp service deterioration; increasing fleet size without first meeting this power level provides limited benefit. Further, to compare levers on a common scale, we compute the elasticity $(\eta )$ for each KPI $Y$ with respect to a lever $X$ as described in (29):

29 $$\eta ({Y|X})=\frac{\Delta Y/{Y}^{{base}}}{\Delta X/{X}^{{base}}}$$

Elasticities are derived directly from the percentage changes reported in the sensitivity scenarios. Table 10 summarizes headline elasticities for the transport-priority and energy-provision cases.

**Table 10:** Elasticity analysis

Case	Lever (ΔX)	KPI	Elasticity (ΔY/ΔX)	Readout
Transport-priority	Charging power (Δ50%)	Passengers	0.27	Charging power is the dominant mobility lever.
	Fleet size (Δ20%)		0.08	Adding vehicles yields small gains at current infrastructure.
	Battery capacity (Δ25%)		0.05	Battery size has marginal effect on throughput.
Energy-provision	Discharge tariff (Δ33%)	Revenue	1.14	Tariff strongly drives operator revenue with little mobility impact.
	Discharging power (Δ108%)	Energy delivered	0.51	Energy output is rate-limited by discharge power.
	Charging power (Δ50%)	Passengers	1.23	Faster turnaround substantially lifts mobility even during outages.

The elasticity outcomes indicate that mobility is charger-bound, passenger throughput responds strongly to charging power but weakly to fleet size or battery capacity, while outage performance is driven by price and power. In the energy-provision case, revenue is highly elastic with respect to the discharge tariff, and delivered energy scales with discharging power, whereas faster charging chiefly restores mobility even during outages.

## Discussion

To unlock the full potential of SAEVs for grid resilience, policies must treat fleets and neighborhood charging as vital public infrastructure. Funding should focus on bidirectional, high-power neighborhood hubs, not just more vehicles. Public capital should be allocated to upgrade interconnections and install V2G-capable connectors in densely populated areas, with streamlined permitting and standardized connections. Grants, green bonds, or utility strategies covering hardware and protection costs will expand energy support and reduce project turnaround times.

Tariff design must ensure attractive pay to encourage participation during disruptions. A good rule is to set the emergency discharge price above the operator’s opportunity cost plus battery degradation cost, with a margin; indexing this to scarcity (residual deficit at feeders) sustains participation during crises and tapers as restoration occurs. To stay prepared without events, policymakers should use a pay-for-availability model (capacity payment per connected kW with verified SOC and dwell time), settled against measured energy and adherence to guardrails. This combines a tariff floor, scarcity indexation, and availability payment to align private incentives with resilience and prevent under-delivery.

Regulatory enablers are essential. Contracts should mandate V2G-ready fleets and hubs, data sharing for queues, SOC, and hub usage, and interoperability standards to facilitate asset sharing. Interconnection procedures should include fast-track options with predefined protections and utility-approved capacity. Cities can incorporate V2G readiness into electrification programs, making neighborhood chargers dual-purpose for use and outages. Operational policies should establish clear guidelines for services and energy use to ensure the system operates efficiently. Basic promises, such as waiting times, trip frequency, minimum SOC, and limits on discharging, can help prevent mobility issues. Spatial policy should prioritize resilience zones with dense housing, health services, and mixed-use buildings to maximize social benefits per energy unit and reduce travel. Geofenced maps may help coordinate with operators and utility companies, potentially speeding up service setup. To promote fairness, the plan might include reserving capacity for essential trips, limiting surge prices during outages, and offering fare supports or service guarantees in lower-income neighborhoods. A practical adoption pathway develops from these elements and can be broken down into three action steps:

1. **Pre-event (Plan and prepare):** Co-site high-power, V2G-capable hubs in designated resilience zones and secure permits early. Establish clear agreements that outline the emergency tariff formula, availability retainers, mobility and SOC guardrails, required data fields, activation thresholds, and verification procedures. Standardize interconnection and communications so different fleets can use the same hubs and run regular tabletop and live-drill tests to validate the playbook.
2. **During event (Activate and operate):** Activate automatically when magnitude or duration thresholds are crossed. Set the emergency price at the guaranteed floor, then adjust it to measured scarcity (residual deficit). Enforce mobility and SOC guardrails while dispatch prioritizes the pre-designated resilience zones; pause or throttle V2G if queues rise or SOC approaches the floor. Refresh decisions every 10–15 min using a minimal data loop, which includes anonymized queues and hub utilization from operators, as well as residual deficit or restoration status from the utility.
3. **Post-event (Settle and learn):** Reconcile payments based on metered kilowatt-hours, verified availability, and adherence to service guarantees. Conduct a concise after-action review and share a performance summary. Apply what you learn to refine resilience-zone maps, activation thresholds, tariff parameters, hub siting, and the terms of the agreements in preparation for the next event.

Finally, these recommendations rely on the assumption of widespread SAEV and V2G infrastructure availability. Near-term policies should fund bidirectional neighborhood hubs, V2G fleet requirements, interoperable standards, and payments that sustain a resilience market. This ensures cities can turn SAEVs into reliable district energy tools without compromising mobility.

## Conclusions

This study examines the operational and economic conditions under which SAEVs can provide both mobility and grid support during urban power outages. Our Montreal case study demonstrates that SAEVs can enhance grid resilience while fulfilling basic mobility needs when system design and operations are carefully managed. The developed optimization experiments reveal a clear trade-off. The transport-only extreme serves 5692 passengers and produces no energy, while the energy-priority extreme supplies 7238 kWh and serves 1556 passengers. SAEVs thus act as neighborhood-scale emergency storage, but their energy role must be limited to prevent significant reductions in mobility, and larger fleets would be needed for severe outages. Battery cycling increases under energy priority, but we explicitly consider degradation, keeping dispatch economically feasible. Additionally, sensitivity analyses identify practical levers for action. For mobility, charging power and availability impact throughput more than adding vehicles or larger batteries, and low-power chargers significantly slow service. For energy provision, revenues grow with fleet size and discharge power, and a higher event tariff improves operator economics with minimal impact on mobility.

We acknowledge the limitations of this study and propose directions for future research. An ε-constraint method is employed to solve the MOO problem, providing exact solutions. However, its computational burden may be impractical for larger instances, suggesting the need for heuristics to find suboptimal solutions and expand this work. A deep analysis of grid dynamics is outside the scope of this study; future work will couple the SAEV optimization with feeder-level power flow, restoration sequencing, and other DERs to co-optimize mobility and grid operations. Also, our analysis represents travel demand deterministically, using observed OD matrices, to construct an exact and comparable Pareto frontier for the mobility–energy trade-off. We recognize that outages can induce demand adaptations through cancellations, deferrals, mode shifts, and slower corridors. A practical next step is to implement a hybrid architecture that couples an agent-based demand layer with the existing outer-loop optimization. This extension is left for future work, given the lack of outage-specific elasticity estimates at the spatial and temporal resolution used here. Future work should also test the framework under more severe and spatially heterogeneous outages, including multi-day events, and incorporate temperature and weather effects on energy use and vehicle performance, for example, through robust optimization over consumption ranges. The analysis should also extend to capital and operating costs to assess long-run profitability.

In conclusion, our findings emphasize the importance of a robust charging infrastructure, ample discharge capacity, and an appealing utility compensation model, enabling operators to advance energy innovation without disrupting essential transportation services. In this context, we believe that the developed MOO model offers a valuable framework for future research into balancing competing needs and ensuring that operational strategies align with SAEV-O’s priorities and constraints for transportation and energy activities.

## Data Availability

The data supporting this study are openly available and referenced in this paper.
